# Physiology guidance for coronary artery disease in patients with severe aortic stenosis: are we there yet?

**DOI:** 10.1093/ehjopen/oeaf051

**Published:** 2025-06-06

**Authors:** Jacob T Lønborg

**Affiliations:** Copenhagen University Hospital, Inge Lehmanns vej 7, Copenhagen 2100, Denmark

**Authors' response to the commentary of Minten and Dubois, https://doi.org/10.1093/ehjopen/oeaf052.**

We thank Dr. Minten and Dubois for a relevant comment regarding the physiology assessment of coronary artery disease in patients with severe aortic stenosis (AS).^1^ In general, we agree with the issues raised. Due to the impact of severe AS on the haemodynamic and the myocardial structure, these patients have increased coronary flow reserve, increased microvascular resistance, and increased resting flow.^2,3^ After aortic valve replacement, the coronary flow reserve increases, the microvascular resistance decreases, the resting flow decreases, but the hyperaemic absolute flow roughly remains unchanged.^2,3^ Due to these physiological changes, the resting indices for assessing coronary artery disease increase significantly after valve replacement, whereas the fractional flow reserve (FFR) decreases slightly or remains unchanged.^2,4^ It has been demonstrated that resting indices change across the cut-off value of 0.89 in around 40% of the patients compared with 10–20% for FFR using a 0.80 cut-off.^2,4^ However, the negative predictive value for resting indices is close to 100%, whereas the positive predictive value for FFR is close to 90%.^2,4^ Therefore, combining a resting index to exclude and FFR to confirm haemodynamic significant coronary artery disease could be a suggested strategy. However, having said this, the NOTION-3 trial showed that the benefit from revascularization was most pronounced in patients a coronary lesion with diameter stenosis ≥90% in whom FFR was not used to assess the lesion,^5^ in agreement with previous observations that the benefit from revascularization correlates with the severity of ischaemia (FFR value).^6^ It may thus be questioned whether it is indicated to perform revascularization of coronary lesions with FFR value in the border zone in patients with severe AS undergoing TAVI—especially in the light of increased bleeding risk.^5,7^

## References

[1] Minten L, Dubois C. Physiology-guided PCI in patients with severe aortic stenosis undergoing TAVI: are we there yet? Eur Heart J Open; doi:10.1093/ehjopen/oeaf052. PMC12142461 40486725

[2] Minten L, McCutcheon K, Vanhaverbeke M, Wouters L, Bézy S, Lesizza P, Jentjens S, Frederiks P, Bringmans T, Voigt JU, Adriaenssens T, Desmet W, Sinnaeve P, Jacobs S, Verbrugghe P, Meuris B, Janssens S, Fearon WF, Bennett J, Dubois C. Coronary physiological indexes to evaluate myocardial ischemia in patients with aortic stenosis undergoing valve replacement. JACC Cardiovasc Interv 2025;18:201–212. 39474985 10.1016/j.jcin.2024.10.024

[3] Sabbah M, Olsen NT, Holmvang L, Tilsted HH, Pedersen F, Joshi FR, Sørensen R, Jabbari R, Arslani K, Sondergaard L, Engstrøm T, Lønborg JT. Long-term changes in coronary physiology after aortic valve replacement. EuroIntervention 2023;18:1156–1164. 36239118 10.4244/EIJ-D-22-00621 PMC9940233

[4] Sabbah M, Joshi FR, Minkkinen M, Holmvang L, Tilsted HH, Pedersen F, Ahtarovski K, Sørensen R, Thue Olsen N, Søndergaard L, De Backer O, Engstrøm T, Lønborg J. Long-term changes in invasive physiological pressure indices of stenosis severity following transcatheter aortic valve implantation. Circ Cardiovasc Interv 2022;15:e011331. 34809440 10.1161/CIRCINTERVENTIONS.121.011331

[5] Lønborg J, Jabbari R, Sabbah M, Veien KT, Niemelä M, Freeman P, Linder R, Ioanes D, Terkelsen CJ, Kajander OA, Koul S, Savontaus M, Karjalainen P, Erglis A, Minkkinen M, Sørensen R, Tilsted HH, Holmvang L, Bieliauskas G, Ellert J, Piuhola J, Eftekhari A, Angerås O, Rück A, Christiansen EH, Jørgensen T, Özbek BT, Glinge C, Søndergaard L, De Backer O, Engstrøm T; NOTION-3 Study Group. PCI in patients undergoing transcatheter aortic-valve implantation. N Engl J Med 2024;391:2189–2200. 39216095 10.1056/NEJMoa2401513

[6] Johnson NP, Tóth GG, Lai D, Zhu H, Açar G, Agostoni P, Appelman Y, Arslan F, Barbato E, Chen SL, Di Serafino L, Domínguez-Franco AJ, Dupouy P, Esen AM, Esen OB, Hamilos M, Iwasaki K, Jensen LO, Jiménez-Navarro MF, Katritsis DG, Kocaman SA, Koo BK, López-Palop R, Lorin JD, Miller LH, Muller O, Nam CW, Oud N, Puymirat E, Rieber J, Rioufol G, Rodés-Cabau J, Sedlis SP, Takeishi Y, Tonino PA, Van Belle E, Verna E, Werner GS, Fearon WF, Pijls NH, De Bruyne B, Gould KL. Prognostic value of fractional flow reserve: linking physiologic severity to clinical outcomes. J Am Coll Cardiol 2014;64:1641–1654. 25323250 10.1016/j.jacc.2014.07.973

[7] Patterson T, Clayton T, Dodd M, Khawaja Z, Morice MC, Wilson K, Kim WK, Meneveau N, Hambrecht R, Byrne J, Carrié D, Fraser D, Roberts DH, Doshi SN, Zaman A, Banning AP, Eltchaninoff H, Le Breton H, Smith D, Cox I, Frank D, Gershlick A, de Belder M, Thomas M, Hildick-Smith D, Prendergast B, Redwood S; ACTIVATION Trial Investigators. ACTIVATION (PercutAneous Coronary inTervention prIor to transcatheter aortic VAlve implantaTION): a randomized clinical trial. JACC Cardiovasc Interv 2021;14:1965–1974. 34556269 10.1016/j.jcin.2021.06.041

